# Invader Competition with Local Competitors: Displacement or Coexistence among the Invasive Khapra Beetle, *Trogoderma granarium* Everts (Coleoptera: Dermestidae), and Two Other Major Stored-Grain Beetles?

**DOI:** 10.3389/fpls.2017.01837

**Published:** 2017-11-07

**Authors:** Nickolas G. Kavallieratos, Christos G. Athanassiou, Raul N. C. Guedes, Johanna D. Drempela, Maria C. Boukouvala

**Affiliations:** ^1^Laboratory of Agricultural Zoology and Entomology, Department of Crop Science, Agricultural University of Athens, Athens, Greece; ^2^Laboratory of Entomology and Agricultural Zoology, Department of Agriculture, Crop Production and Rural Environment, University of Thessaly, Volos, Greece; ^3^Departamento de Entomologia, Universidade Federal de Viçosa, Viçosa, Brazil; ^4^Laboratory of Organic Chemistry, Department of Chemistry, University of Ioannina, Ioannina, Greece

**Keywords:** insects’ competition, species displacement, competitive exclusion, species co-existence, storedproducts, quarantine species

## Abstract

Local potential competitor species are important determinants of the invasibility of an environment even when widely recognized invasive species are concerned since it may compromise its establishment. Thus, the outcome of the direct competition among the invasive khapra beetle, *Trogoderma granarium*, and the cosmopolitan species lesser grain borer, *Rhyzopertha dominica* and rice weevil, *Sitophilus oryzae*, and thus the likelihood of establishment of *T. granarium* under their co-occurrence, was here explored in paddy rice and wheat, at temperatures between 25 and 35**°**C and through 200 days of storage. Insect infestations were higher in wheat rather than in paddy rice. *Trogoderma granarium* was unable to displace any of the competing species under two and three-species competition experiments retaining lower adult population than both local competitors at the lowest temperature level. *Rhyzopertha dominica* prevailed in paddy rice, while *S. oryzae* prevailed in wheat. Paradoxically, *T. granarium* adults retained low population growth but contributed more for the total frass production and grain loss, much more than that recorded for *R. dominica*. Nonetheless, *T. granarium* larvae exhibited high population numbers 130 days after the introduction of the parental individuals. At higher temperature levels (30 and 35**°**C) the numbers of *T. granarium* larvae were extremely high even after 65 days, while the numbers of the other two species rapidly declined. Interestingly, the simultaneous presence of *R. dominica* and *S. oryzae* was beneficial for the population growth of *T. granarium*. Consequently, *T. granarium* has the ability to outperform other primary stored-product insects at high temperatures, while its presence at low temperatures remains for long periods apparently unaffected by other co-occurring species. Hence, *T. granarium*, in wheat, is able to outcompete other major species of stored-product insects at elevated temperatures, while at 25**°**C this species can maintain low numbers of individuals for long periods, which can rapidly produce population outbursts when the prevailing conditions are suitable for its development.

## Introduction

Invasive species are those established outside their native range resulting in potential or realized impact in the invaded environment (Hill et al., 2016). Such impact of an invasive insect species is usually regarded as due to its economical implications, particularly referring to its control, or to direct effects in biodiversity (Hill et al., 2016). Indeed, overall global costs of biological invasions are voluminous, even if they are often underestimated (Bradshaw et al., 2016), and configure to a standard expectation for pest-risk analysis and decision-making to recognize their quarantine relevance and status (Soliman et al., 2015; Food and Agriculture Organization of the United Nations [FAO], 2016; Liebhold et al., 2016; European and Mediterranean Plant Protection Organization [EPPO], 2017). However, whilst these (direct) impacts remain the core concern of biological invasions, the concerns of some indirect impacts of invasive species are still poorly understood (Hawkins et al., 2015; McGeoch et al., 2015; Kumschick et al., 2016). Some of these indirect impacts, including effects on food-webs and species assemblages, are dependent on drivers of the processes of establishment and spread of invasive species, which together with the arrival and eventual impact encompasses the four conceptual stages of the invasions, i.e., arrival, establishment, spread, and impact (Lockwood et al., 2013; Hill et al., 2016).

Great credence is laid on the arrival and potential (direct) impact of invasive species, which is largely the result of international trade, transportation, and recreation (Yemshanov et al., 2014; Banks et al., 2015), potentially aggravated by the current scenario of global climate change, which usually favors the spread and establishment of species better adapted to warmer temperatures (Kriticos et al., 2013; Jung et al., 2016). As a consequence, modeling the international trade and transport pathways to improve biosecurity surveillance can be largely aided by climate change models, while these models can be also utilized in designing eradication strategies and regulatory initiatives regarding biological invasions (Colunga Garcia et al., 2013; Kriticos et al., 2013; Yemshanov et al., 2014; Douma et al., 2016; Liebhold et al., 2016). The establishment and subsequent spread of invasive species in general, or invasive insect species in particular, depend on its invasiveness potential and the environmental context of the invasion, which determines the suitability and thus the “invasibility” of a given environment (Duncan, 2016; Hui et al., 2016). While the former is recognized by the invader rate of population growth after the initial propagule introduction, the latter is a property of the environment determining its vulnerability to invasion (Hill et al., 2016; Hui et al., 2016). However, both characteristics, the species invasiveness and the local context where such invasion may take place, are interdependent and should be considered together.

The khapra beetle, *Trogoderma granarium* Everts (Coleoptera: Dermestidae) is a long standing quarantine species in major parts of the world, while it is recognized among the world’s 100 most important invasive species (Banks, 1977; Lowe et al., 2000; Myers and Hagstrum, 2012). From its likely origin in the Indian subcontinent, as recorded in the late 1800’s, *T. granarium* has spread to several countries in Asia, and subsequently to Middle East, Africa, and Europe, through transportation of durable commodities (Stibick, 2009; Paini and Yemshanov, 2012; Day and White, 2016). The species is recognized as an A2 quarantine species (European and Mediterranean Plant Protection Organization [EPPO], 1981, 2013, 2017), and subjected to strict quarantine legislation in the Americas and Australia (Stibick, 2009; Myers and Hagstrum, 2012; Day and White, 2016). Among these countries, Australia, Brazil, Canada, and the United States are major exporters of durable agricultural commodities, such as wheat, and likely the foremost countries with quarantine restrictions against this species (Stibick, 2009; Myers and Hagstrum, 2012; Day and White, 2016; Ministério da Agricultura Pecuária e Abastecimento [MAPA], 2016).

*Trogoderma granarium* does particularly well in stored cereals and cereal products, especially at warm and dry conditions, but it has been recorded in about 100 different commodities, including products of both animal and plant origin (Finkelman et al., 2006; Hosseininaveh et al., 2007; Burges, 2008; Hagstrum and Subramanyam, 2009). This species is considered a “dirty feeder” because it damages more grain kernels than it actually consumes, contaminates grain and grain products with body parts and setae, and severe infestations make the grain and grain products unpalatable and unmarketable justifying quarantine concern in exporters of agricultural commodities (Stibick, 2009; Myers and Hagstrum, 2012). Furthermore, *T. granarium* exhibits refuge seeking behavior and long facultative diapause, which can last for 6 years or more, making its detection and, especially control, extremely difficult (Bell and Wilson, 1995; Stibick, 2009; Wilches et al., 2016). Its diapausing larvae are particularly tolerant to different chemical and non-chemical methods, including extreme temperatures and fumigants (Bell and Wilson, 1995; Wilches et al., 2016). The adults of this species do not fly and hence, diapausing larvae, which often remain undetected, are considered the life stage that contributes to its spread in different eco-zones (Banks, 1977). In fact, even the non-diapausing larvae have been proved tolerant to many of the currently used insecticides at dose rates that are effective to other major stored-product insect species (Kavallieratos et al., 2016, 2017). In this context, considering the difficulties in controlling this species, the potential establishment and spread of *T. granarium* is also directly related with the invasibility of the environment, including the temperature and humidity conditions, as well as local competitors (Aitken, 1975).

The likelihood of *T. granarium* invasion in Canada is considered low, except in heated storage facilities (Wilches et al., 2016), in contrast with the southwestern United States, most of Australia and Brazil (Banks, 1977; Stibick, 2009; Day and White, 2016; Ministério da Agricultura Pecuária e Abastecimento [MAPA], 2016). Nevertheless, in any potential introduction and establishment in new areas, *T. granarium* will have to face and compete successfully against local competitors that may share the same environment and have the potential to compromise its eventual establishment and further spread. However, paradoxically, despite the number of studies on the biology of *T. granarium*, the issue of competition has yet to be considered. In this regard, the relatively limited presence of this species in some areas, such as the Mediterranean Europe might be, at least in part, due to the presence of co-occurring beetle species of stored cereals, including the lesser grain borer, *Rhyzopertha dominica* (F.) (Coleoptera: Bostrychidae), and the rice weevil, *Sitophilus oryzae* (L.) (Coleoptera: Curculionidae), which are also key pest species of stored cereals and cereal products in the Americas and Australia (Aitken, 1975; Rees, 1996; Opit et al., 2012). Although also invasive species, these two stored-product insect pests currently exhibit cosmopolitan distribution spanning different climatic zones, often causing devastating infestations in several types of grains (Hagstrum and Subramanyam, 2009). In contrast with *T. granarium*, whose larvae are external feeders and immature development occurs outside the grain kernel, the immatures of both *R. dominica* and *S. oryzae* are exclusively internal feeders and remain within the grain kernel throughout their development until reaching the adult stage (Aitken, 1975; Rees, 1996).

As newly introduced *T. granarium* individuals are likely to face the competition of other major species that share the same habitat, such as *R. dominica* and *S. oryzae*, their interaction under competition is a potentially important determinant to the establishment of this invasive species. Nonetheless, these three species explore different ecological niches within the same habitat with *S. oryzae* laying eggs within the grain kernel, while *R. dominica* lay eggs outside the grain kernel and the larvae penetrates the grain in which it develops, both as internal feeders, in contrast with *T. granarium* whose larvae are long-lived external feeders (Aitken, 1975; Hill, 2003); such distinct life histories allow for potential resource partition allowing co-existence of at least two species. Regardless, competition is recognized as an important phenomenon among grain beetles driving their natural selection and population regulation (Smith and Lessells, 1985; Smith, 1990, 1991), and has been tested in few species so far, but never in the case of *T. granarium* (Nicholson, 1954; De Jong, 1976; Connell, 1983; Krebs, 2002). Moreover, despite the considerable number of studies that are available for competition in stored-product beetles, such studies were usually focused in intraspecific competition, by conducting parallel experiments of life table characteristics of single species (Smith and Lessells, 1985; Smith, 1990; Horn, 1997; Guedes et al., 2010; Oliveira et al., 2015), and not interspecific competition (i.e., simultaneous presence of different species in the same substrate). The outcome of this competition is apparently determined by several biotic and abiotic conditions, including temperature and type of commodity, but there is still inadequate information regarding the effect of these conditions in competition of *T. granarium* with other major stored-grain insects. Therefore, a series of direct competition experiments involving *T. granarium*, *R. dominica*, and *S. oryzae* was performed, to shed light toward this direction, as an aid to understand the role of local competitors in *T. granarium* establishment and spread.

The competition experiments involving *T. granarium*, *R. dominica*, and *S. oryzae* were performed under three temperature conditions (25, 30, and 35°C) and in two major cereal host species, paddy rice and wheat, to assess the likelihood of establishment or displacement of these potentially co-existing and competing species, their potential relative prevalence and eventual grain loss. As *T. granarium* is better adapted to cereals and warmer temperatures (Banks, 1977; Finkelman et al., 2006; Hosseininaveh et al., 2007; Burges, 2008), we hypothesized that *T. granarium* is more likely to prevail at the highest temperature, unlike *R. dominica* and *S. oryzae*, which originate and are better adapted to temperate and subtropical conditions requiring lower temperatures for their development (Potter, 1935; Fields et al., 1993; Guedes et al., 1997; Corrêa et al., 2013, 2017). For this purpose, we proceeded with the experimental assessment of these scenarios, in order to further evaluate the invasion risks and prevalence of *T. granarium* in new eco-zones.

## Materials and Methods

### Insects

The three species of stored-grain beetles used were obtained from laboratory colonies of *R. dominica* and *S. oryzae* initially collected from Greek storage facilities by 2006 and are currently maintained at the Laboratory of Agricultural Zoology and Entomology of the Agricultural University of Athens, Greece. The colony of *T. granarium* was established in 2014 from insects collected in a Greek storage facility and since then has also been maintained in the same Laboratory. *Rhyzopertha dominica* and *S. oryzae* were cultured in whole wheat at 27°C, 65% relative humidity and continuous darkness. *Trogoderma granarium* was cultured in whole wheat at 30°C, 65% relative humidity and continuous darkness.

### Commodities

Grains of two commodities were used in the experiments, rough (paddy) rice (*Oryza sativa* L. var. Thaibonnet), and hard wheat (*Triticum durum* Desf. var. Claudio). Prior to experimentation, the grain moisture content was adjusted to 13.5 ± 0.5%, as determined by a moisture meter (mini GAC plus, Dickey-John Europe S.A.S., Colombes, France). Thus, grains were dried inside an oven at 50°C or by adding distilled water according to their initial moisture content (Kavallieratos et al., 2012; Vassilakos et al., 2015).

### Bioassays

#### General Procedures

Three competition experiments were carried out with the three beetle species. One exploring intra-species competition for each (isolated) species, one with two species in all three possible combinations, and a last experiment with three species together. All three sets of competition experiments were performed using both commodities (i.e., paddy rice and wheat), three temperatures (25, 30, and 35°C), and three storage periods (65, 130, and 200 days of infestation) at 65% relative humidity in environmentally controlled laboratory facilities. Each treatment for each insect combination was replicated nine times.

The experimental units were cylindrical glass vials (12 cm high × 7 cm in diameter) containing 20 g of grain (either paddy rice or wheat), as determined using a Precisa XB3200D compact balance (Alpha Analytical Instruments, Gerakas, Greece). The lids of the vials had a 1.5 cm diameter opening that was covered by muslin gauze to allow ventilation and the upper inner walls were coated with polytetrafluoroethylene (PTFE 60 wt % dispersion in water; Sigma–Aldrich Chemie GmbH, Taufkirchen, Germany) to prevent the insects from escaping.

#### Treatment Combinations

A fixed number of 15 unsexed insects of each species (<2 weeks old adults of *R. dominica* and *S. oryzae*, and <24 h old adults of *T. granarium*) were placed in each vial. Thus, in the bioassays with single species 15 (unsexed) adult insects of a given species were placed in each vial, and the vials were placed at the different conditions of host, temperature and storage period indicated previously. Fifteen adults of each species were also used for the interspecific competition experiments involving two species contemplating the combinations *R. dominica* + *S. oryzae, S. oryzae* + *T. granarium*, and *T. granarium* + *R. dominica.* The last experiment with the competition among all three species was set up using again 15 adult insects of each species placed in each vial containing 20 g of grain, as above. These insect densities are relatively high and they were used to allow for competition to take place. The vials were then placed at the conditions described previously, and were opened after 65, 130, and 200 days. Subsequently, the total numbers of (live and dead) individuals within each vial were recorded. Given that *R. dominica* and *S. oryzae* are internal feeders, only adults were recorded, while in the case of *T. granarium* adults, larvae and pupae were also recorded. Since the vast majority of these immatures were at the larval stage (number of pupae was low), only larvae were considered in the analysis. There were different sets of vials for each of the storage periods tested. After insect counting, the grains from each vial were sieved and the amount of frass produced, as well as the kernels, were weighted separately using a Precisa XB3200D compact balance. Grain loss was determined as final grain weight (%) [i.e., {initial grain weight (20 g) - [weight of frass (g) + weight of kernels (g)]}× 100/initial grain weight (20 g)] (Padín et al., 2002).

### Statistical Analyses

The results of the numbers of adults were subjected to a four-way analysis of variance for the single-species experiment (insect species, host, temperature, and time), and to three-way analyses of variance (host, temperature, and time) for each combination of species in the experiment of two competing species, always using the GLM procedure from SAS (SAS Institute, 2008). The results of frass produced and grain loss obtained in these two experiments were subjected to a two-way analysis of variance (host × insect species) with temperature and time as covariate since there was no need to test the direct effect of these covariates for these responses, which are the result of insect activity on host grains (PROC GLM) (SAS Institute, 2008). The relationships between insect infestation and frass produced, and grain loss, were tested using correlation analyses (PROC CORR) (SAS Institute, 2008).

The three-species competition results of adult insect numbers from each species, overall frass produced and grain loss were subjected to a three-way multivariate analysis of variance to test if there is overall effect of host, temperature, and time (PROC GLM with MANOVA statement) (SAS Institute, 2008). Subsequent (univariate) analyses of variance were performed for each set of results recorded, when appropriate (PROC GLM) (SAS Institute, 2008). The number of *T. granarium* larvae produced in each competition scenario was subjected to a four-way analysis of variance with host, competition scenario, temperature, and time as independent variables (PROC GLM) (SAS Institute, 2008). Frass produced and grain loss were subjected to regression analyses with the final insect infestation as independent variable and using the curve-fitting procedure of TableCurve 2D (Systat Software, 2013). The assumptions of normality and homoscedasticity were checked before each analyses (PROC UNIVARIATE) (SAS Institute, 2008); no data transformation was necessary.

A path analysis was used to summarize the general trends and effects of the experiments with two and three simultaneously competing species; this hierarchical statistical analysis structured as a path diagram was performed following guidelines provided by Mitchell (1993). Thus, the effect of both hosts after 200 days of infestation was considered while testing the effect of temperature and inter-species competition in the (adult) population growth of each species and consequent frass produced and grain loss. The instantaneous rate of population growth (*r*_i_) of each species, a robust estimator of population growth (Walthall and Stark, 1997; Stark and Banks, 2003), was calculated using the formula *r*_i_ = [Ln (*N*i/*N*f)]/Δ*t*, where *N*i and *N*f are the initial and final number of live adult insects (in each vial), respectively, and Δ*t* is the storage period (i.e., 200 days). The path analysis was performed using the procedures PROC REG and PROC CALIS from SAS (SAS Institute, 2008).

## Results

### Single-Species Experiment

**FIGURE 1:**
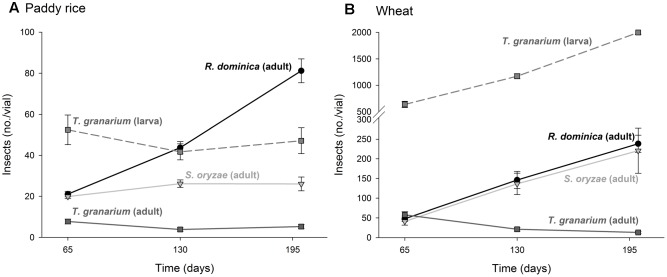
Increase in insect populations from single-species experiment through time in grains of two cereal host species, paddy rice **(A)** and wheat **(B)**. The symbols indicate the average of nine replicates and the vertical bars represent standard errors of the mean.

The four-way analysis of variance for adult progeny produced of each insect species under intra-specific competition was significant (*F* = 56.61, *P* < 0.001, *DF* = 53, 432) and each source of variation tested (i.e., insect species, host, temperature, and time) was also significant, as well as their interactions (*P* < 0.001) (Supplementary Table 1). The intent was to verify if the population growth of each species would respond differently to the host, temperature, and time conditions, what was expected based on their distinct origins and preferences. Wheat favored higher populations for all three species, but the host differences for *T. granarium* were mild and declined with time, unlike those of *R. dominica* and *S. oryzae* (**Figure 1**). The latter did particularly well in wheat with its population increasing with time in this host grain, matching the levels of *R. dominica*, but not in paddy rice.

**FIGURE 2:**
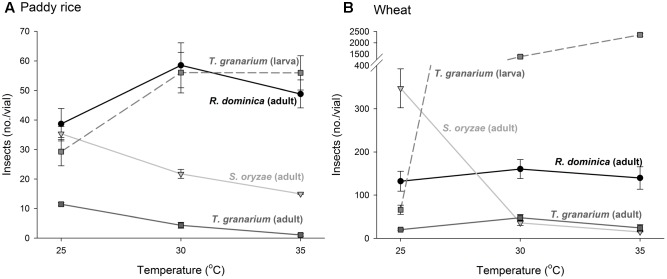
Insect population from single-species experiment at three temperature conditions in grains of two cereal host species. Paddy rice **(A)** and wheat **(B)**. The symbols indicate the average of nine replicates and the vertical bars represent standard errors of the mean.

Temperature increase from 25 to 35°C was detrimental to the adult progeny of *S. oryzae* and *T. granarium* in paddy rice, while *R. dominica* exhibited a peak in population at 30°C and significantly higher adult population numbers at the two higher temperatures (i.e., 30 and 35°C) (**Figure 2A**). The population growth in wheat was distinct with *S. oryzae* maintaining very high population levels at the lower temperature, with a noticeable reduction at higher temperatures (**Figure 2B**). The adult numbers of *R. dominica* and *T. granarium* varied little with temperature in wheat with the later species always exhibiting lower population (<50 adult insects/vial).

**FIGURE 3:**
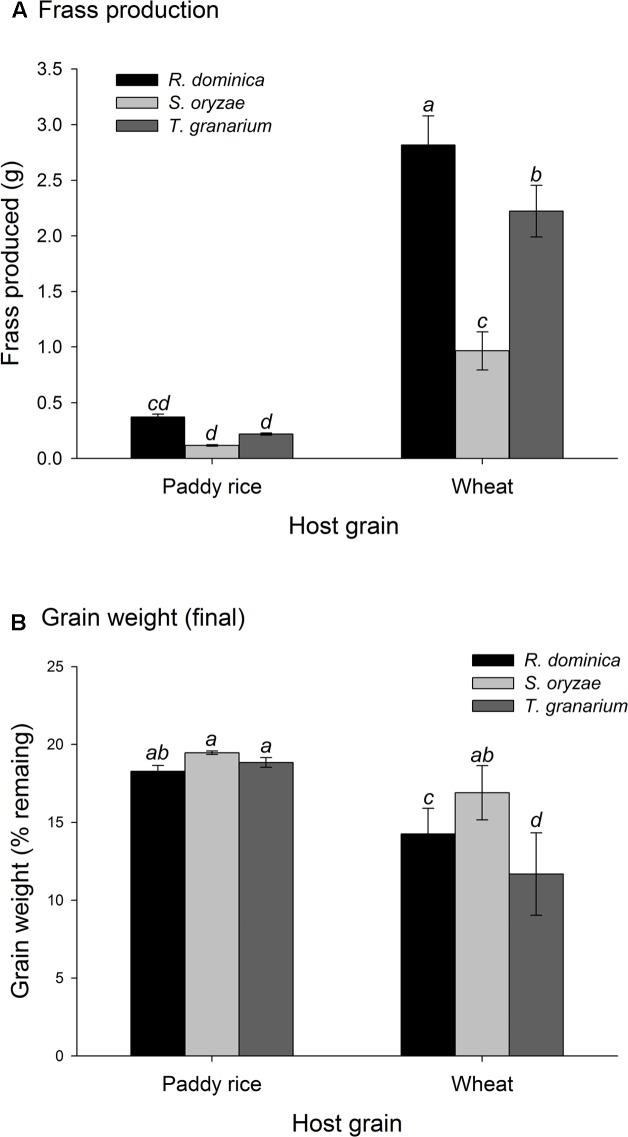
Frass production **(A)** and final grain weight (%) **(B)** by insect populations from single-species experiment in grains of two cereal host species, paddy rice and wheat. The histogram bars refer the average of nine replicates and the vertical bars represent are standard errors of the mean. Histogram bars with the same letter are not significantly different by Tukey’s HSD test (*P* < 0.05).

There was a significant positive correlation between insect population and the amount of frass produced (*n* = 486, *r* = 0.57, *P* < 0.001), and a negative correlation between insect population and grain weight (*n* = 486, *r* = -0.49, *P* < 0.001). As insect infestation and host species are the direct determinants of frass production and grain loss, only insect species and host were tested in the analyses of variance of these response variables using time and temperature as covariates; significant effect of insect species and host were expected. The analyses of variance for frass produced and grain loss using temperature and time as covariates were also significant (frass produced: *F* = 43.35, *P* < 0.001, *DF* = 9, 476; grain loss: *F* = 37.39, *P* < 0.001, *DF* = 9, 476), although the effect of temperature was negligible in both instances (*F* < 1.37, *P* > 0.25, *DF* = 2, 476), unlike the effects of time, besides insect species, host, and their interaction (*P* < 0.001) (Supplementary Table 2). Frass production was always higher in wheat regardless of insect species, but with *R. dominica* followed by *T. granarium* producing significantly more frass than *S. oryzae* (although the differences were negligible in paddy rice) (**Figure 3A**). Grain loss, however, was higher in wheat with *T. granarium* leading to higher losses followed by *R. dominica* and *S. oryzae* (**Figure 3B**). In contrast, paddy rice losses were smaller, regardless of the insect species, with no significant differences (**Figure 3B**).

### Two-Species Competition

**FIGURE 4:**
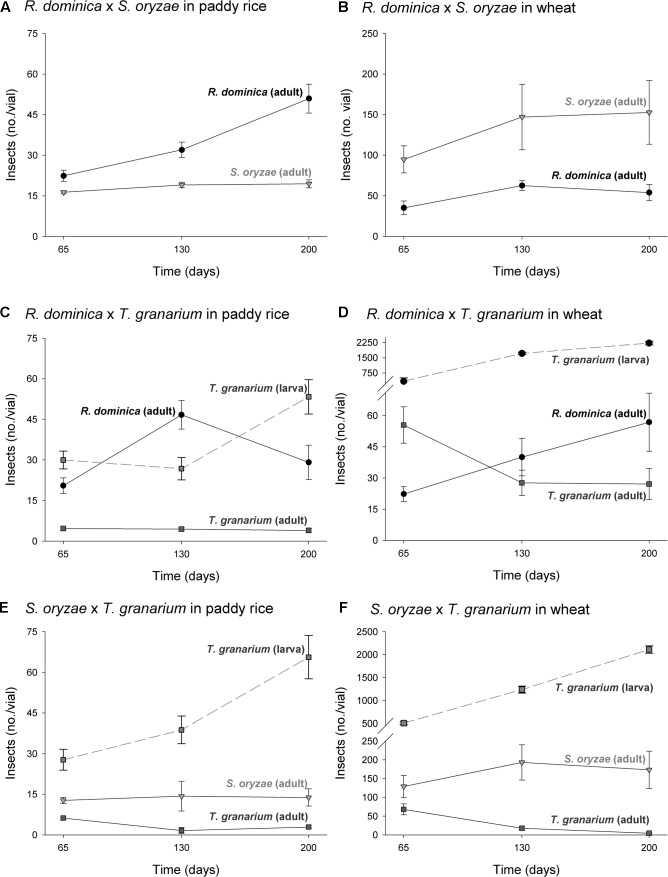
Increase in insect populations under two-species competition through time in grains of two cereal host species, paddy rice and wheat. The symbols indicate the average of nine replicates and the vertical bars represent standard errors of the mean. The labels **(A–F)** refer to each binary group of competing species in two host grains, i.e., paddy rice **(A,C,E)** and wheat **(B,D,F)**.

**FIGURE 5:**
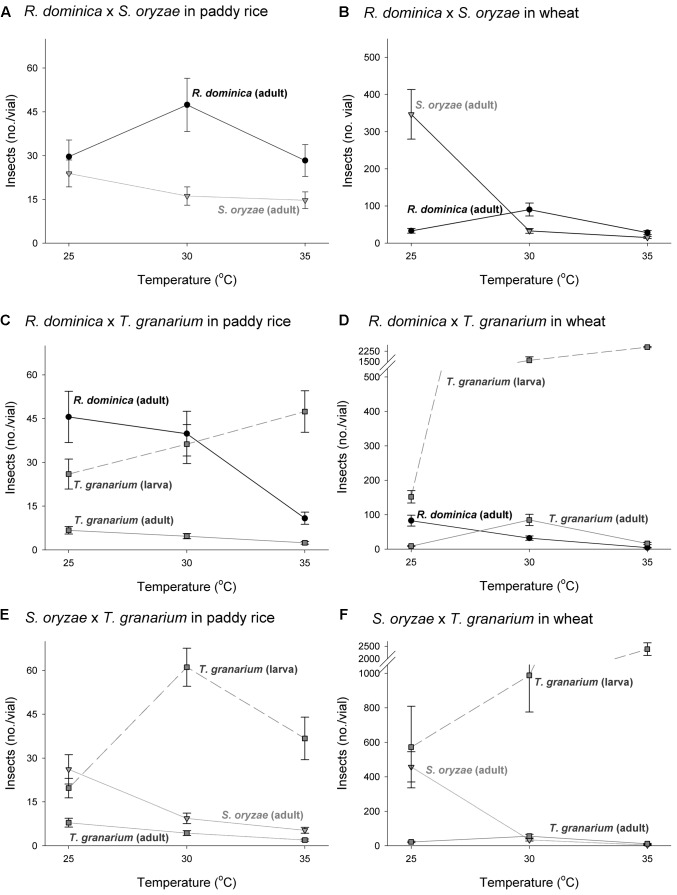
Insect population under two-species competition at three temperature conditions in grains of two cereal host species, paddy rice and wheat. The symbols indicate the average of nine replicates and the vertical bars represent standard errors of the mean. The labels **(A–F)** refer to each binary group of competing species in two host grains, i.e., paddy rice **(A,C,E)** and wheat **(B,D,F)**.

The three-way analyses of variance (host, temperature, and time) for each combination of competing species in the experiment of two species also provided significant differences in the adult progeny production of each species of the competing pair for every pair of competing species (*F* > 10.54, *P* < 0.001, *DF* = 17, 144). As in the single-species experiment, the intent was to verify if the population growth of each species would respond differently to the host, temperature, and time conditions, what was expected based on their distinct origins and preferences. The sources of variation tested, namely host, temperature, and time, besides of their interactions, were also significant in each case (*P* < 0.05) (Supplementary Table 3). Regardless of the competing species and time and temperature conditions, adult insect populations were higher in wheat rather than in paddy rice, and even more so for *S. oryzae* (**Figures 4**, **5**). While *R. dominica* prevailed over *S. oryzae* in paddy rice, the reverse took place in wheat with the latter species exhibiting even stronger prevalence at the lowest temperature tested (i.e., 25°C) (**Figures 4A,B**, **5A,B**). The adults of *R. dominica* also prevailed over the adults *T. granarium* when in paddy rice (**Figures 4C**, **5C**), but such prevalence remained stronger only at the lowest temperature and at longer periods of storage because of a peak in *T. granarium* adults in wheat at 30°C (**Figures 4D**, **5D**). *Sitophilus oryzae* prevailed over *T. granarium* adults in both paddy rice and wheat lasting throughout the storage period (**Figures 4E,F**), but at the lowest temperature (25°C) there was no clear prevalence of *S. oryzae* at 30 and 35°C, considering the adult numbers alone (**Figures 5E,F**).

**FIGURE 6:**
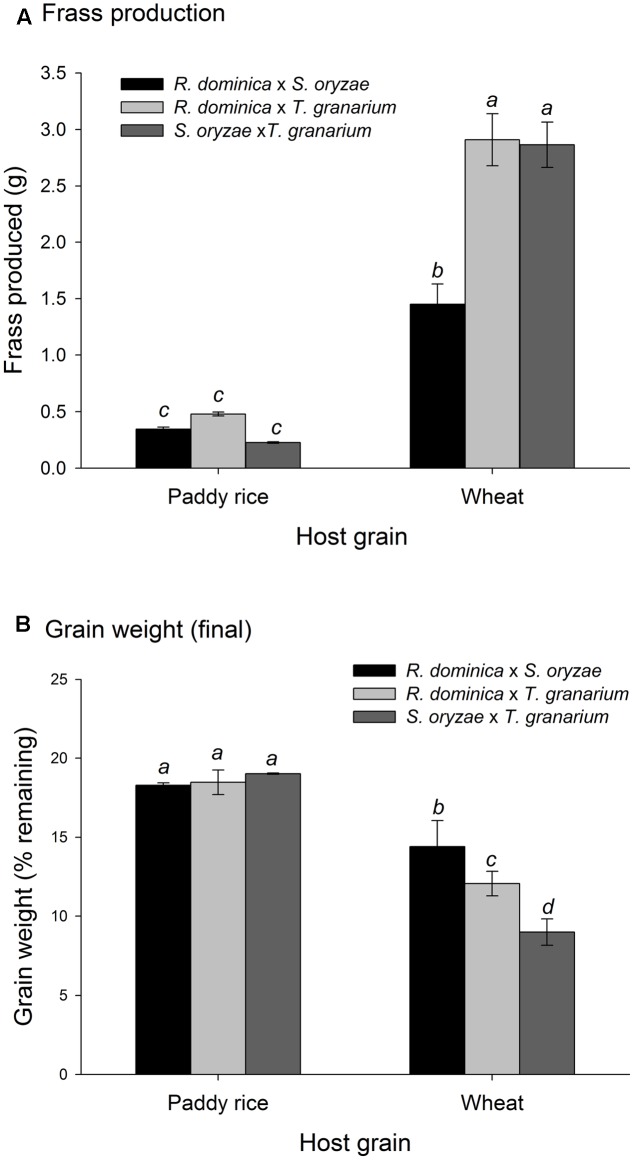
Frass production **(A)** and final grain weight (%) **(B)** by insect populations under two-species competition in grains of two cereal host species, paddy rice and wheat. The histogram bars refer the average of nine replicates and the vertical bars represent standard errors of the mean. Histogram bars with the same letter are not significantly different by Tukey’s HSD test (*P* < 0.05).

Temperature and time were used as covariates in the analyses of variance for frass produced and grain loss in the two-species competition experiments, as in the single-species experiment, because they are not direct determinant of these responses, unlike the insects and hosts. The analyses of variance for frass produced and grain loss using temperature and time as covariates were also significant (frass produced: *F* = 67.45, *P* < 0.001, *DF* = 9, 476; grain loss: *F* = 58.25, *P* < 0.001, *DF* = 9, 476), although the effect of temperature was negligible for frass production (*F* = 1.95, *P* = 0.14, *DF* = 2, 476) (Supplementary Table 4). As for intra-specific competition, frass production was higher in wheat than in paddy rice (**Figure 6A**) without significant difference among the competing pairs for the later. The competition between *S. oryzae* and *T. granarium* led to lower frass produced in wheat than in competition pairs involving *R. dominica*. In contrast, weight grain loss was lower in paddy rice than in wheat with similar losses for each competing pair of species (**Figure 6B**). Nonetheless, the competing pairs involving *R. dominica* led to lower wheat loss and even more so when this species was present with *S. oryzae* (**Figure 6B**).

### Three-Species Competition

**FIGURE 7:**
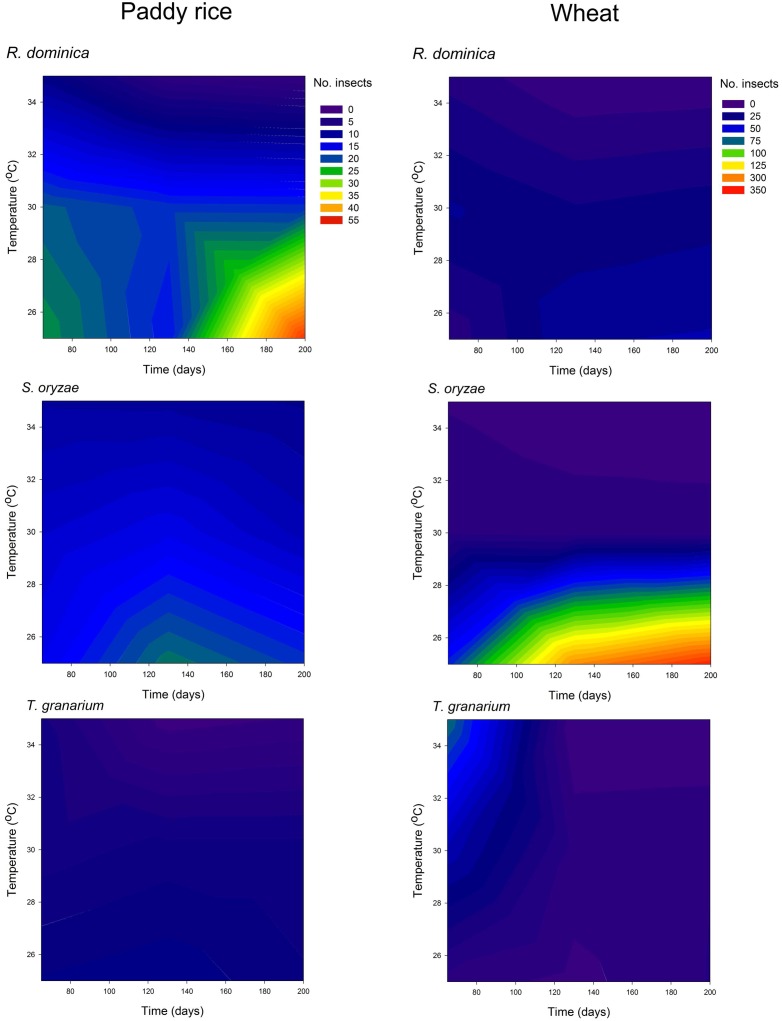
Filled contour plots exhibiting the effect of storage time and temperature on the adult population of three competing species in grains of two cereal host species, paddy rice and wheat.

**FIGURE 8:**
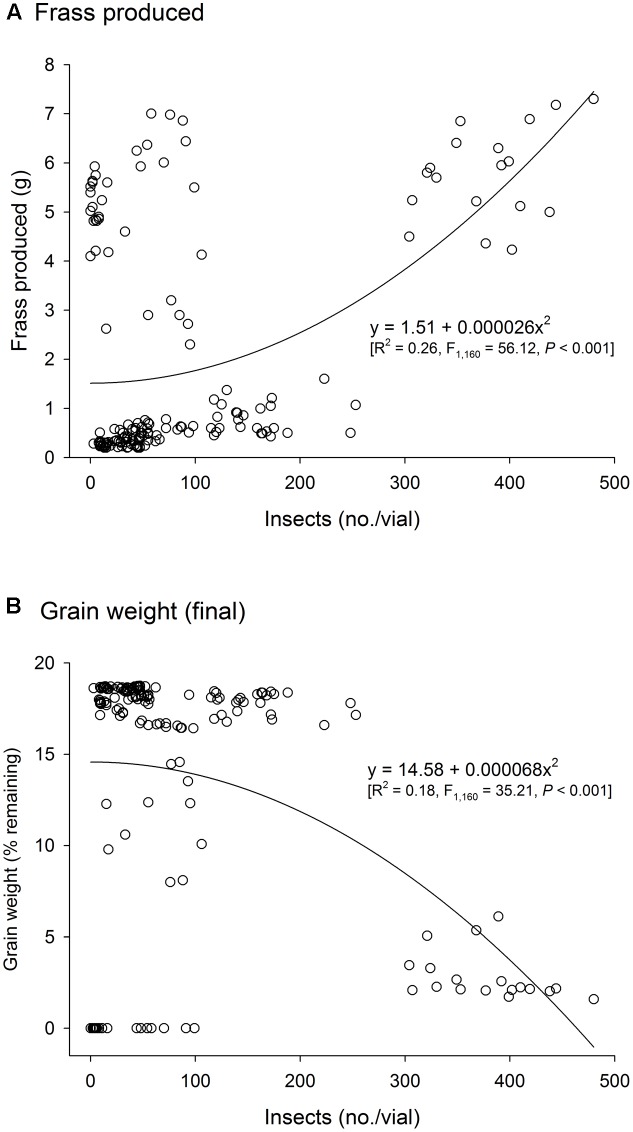
Frass production **(A)** and final grain weight (%) **(B)** produced by three competing species of stored-grain insects, *Rhyzopertha dominica*, *Sitophilus oryzae*, and *Trogoderma granarium*. The symbols represent the observed determinations.

The three-species competition was designed to allow recognition of the likely effects of host, temperature, and time in the population growth of the different species under direct competition, which was expected to take place. The multivariate analyses of variance used to test if there were significant overall differences in the adult progeny produced of each species, frass produced, and grain loss, indicated significant differences for all sources of variation tested (i.e., host, temperature, and time), and their interactions (Wilks’ lambda < 0.049, *F*_aprox_ > 34.59, *P* < 0.001). Such a trend was confirmed in the individual (univariate) analyses of variance performed for each characteristic assessed (Supplementary Table 5). The general trends for the adult population of each species in the three-species competition experiment indicate the persistence of all of them until the 200 days of the experiment with higher populations in wheat than in paddy rice (**Figure 7**). The adult population of *T. granarium* was always smaller than the other two species and remained more stable through time, unlike *R. dominica* and *S. oryzae*, both of which exhibited higher populations with time, particularly at intermediate to lower temperatures for the former in paddy rice, and lower temperatures for the later in wheat (**Figure 7** and Supplementary Table 6). Frass production and grain loss were both significantly affected by the adult insect population present, regardless of the insect species (**Figure 8**).

### Overall Trends on Species Competition and Losses

**FIGURE 9:**
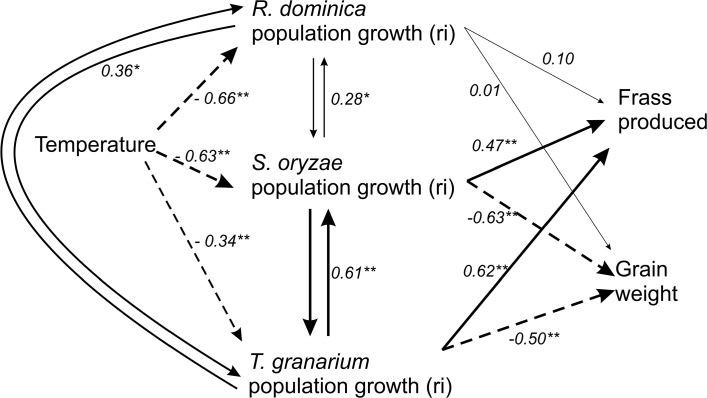
Path analysis diagram for the influence of temperature and interspecific competition on the population growth of three stored-grain insects (*R. dominica*, *S. oryzae*, and *T. granarium*), the frass and grain loss generated. One-headed arrows indicate causal relationships (regression), while double-headed arrows indicate correlation between variables. Significance levels are represented by asterisks (^∗^*P* < 0.05; ^∗∗^*P* < 0.01), and the thickness of each line is proportional to the strength of the relationship. Solid arrows indicate positive relationships, while dashed arrow lines indicate negative relationships. Direct, indirect, and total values for path coefficients are fully presented in **Table 1**.

The relative dominance among competing species and their associated injury were tracked and summarized using a hierarchical approach structured as a path diagram subjected to path analysis (**Figure 9**). Temperature and competing species were expected to affect the adult population growth of the co-existing species, which would determine frass production and grain loss. Temperature directly compromised the adult population growth rate of each competing species, but the strength of such effect was generally smaller for *T. granarium* (**Figure 9** and **Table 1**). Each species was also directly affected by the other competing species in positive associations, in addition to the temperature (**Figure 9** and **Table 1**). In contrast, the main direct determinants of frass production and grain loss were the populations of *S. oryzae* and *T. granarium*, providing strong relationships for both of these grain characteristics (i.e., presence of frass and direct weight loss), unlike *R. dominica* (**Figure 9** and **Table 1**).

**Table 1:** Direct (DE), indirect (IE), and total (TE) effects in the path diagram of **Figure 9** for the model on the influence of temperature and competitor species on the insect population growth, frass production, and grain loss.

Variable	*Rhyzopertha dominica* r_i_			*Sitophilus oryzae* r_i_			*Trogoderma granarium* r_i_			Frass (g)			Grain weight (%)		
	
	DE	IE	TE	DE	IE	TE	DE	IE	TE	DE	IE	TE	DE	IE	TE
Temperature (°C)	-6.88 ×10^-4^	2.45 ×10^-4^	-9.33 ×10^-4^	-1.03 ×10^-3^	-0.18 ×10^-3^	-1.21 ×10^-3^	-0.46 ×10^-4^	-4.63 ×10^-4^	-5.09 ×10^-4^	-	-0.11	-0.11	-	0.47	0.47
*R. dominica r*_i_	-	0.03	0.03	-0.09	-0.16	0.07	-	-	0.30	62.45	68.58	6.13	338.89	-124.00	214.89
*S. oryzae r*_i_	-0.26	-0.01	-0.27	-	-0.07	0.07	0.16	-0.07	0.09	47.62	41.31	87.93	-510.19	-160.08	-670.27
*T. granarium r*_i_	0.12	-0.14	0.02	0.52	0.05	0.57	-	0.10	0.10	219.97	46.94	266.91	-322.64	-316.95	-639.59
*R*^2^		0.82			0.56^∗^			0.63			0.59^∗^			0.63	
*P*		<0.001^∗^			<0.001^∗^			<0.001^∗^			<0.001^∗^			<0.001^∗^	

*The asterisk indicates significant difference at *P* < 0.05*.

### Larval Population of *T. granarium*

**FIGURE 10:**
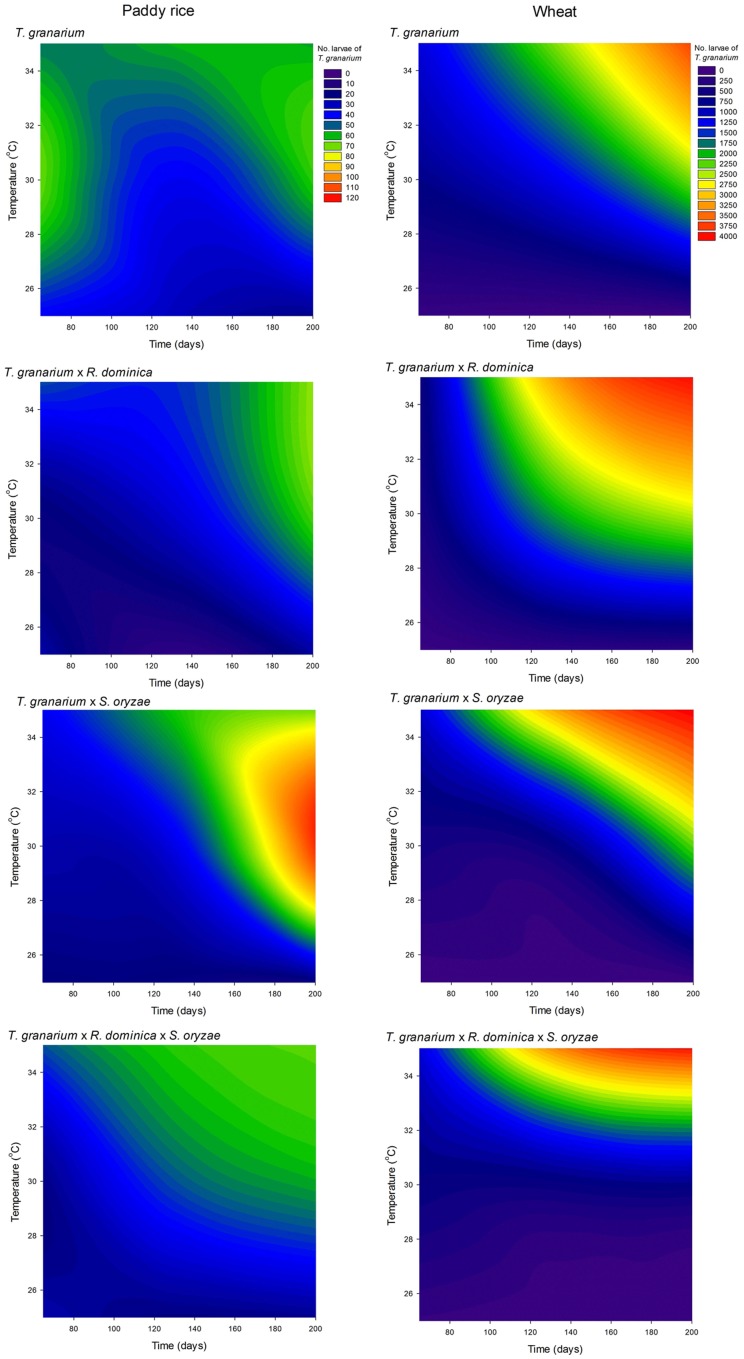
Filled contour plots exhibiting the effect of storage time and temperature on the larva population of *T. granarium* in grains of two cereal host species, paddy rice and wheat, and under different competition scenarios.

As noted above, *T. granarium* larvae are apparent grain contaminants and easily recorded as external feeders, unlike the larvae of the other two species and thus, they were also recorded and analyzed in each competition scenario. *Trogoderma granarium* larvae exhibited much larger numbers than the adults of this species, especially in wheat (**Figure 10** and Supplementary Table 7). The analysis of variance performed was significant (*F* = 497.09, *P* < 0.001, *DF* = 71, 576) with significant effect of all independent variables tested (i.e., host, competition scenario, temperature, and time) in the population of *T. granarium* larvae (*P* < 0.001). High temperatures and longer periods of storage were significantly more favorable to higher populations of *T. granarium* larvae, particularly in wheat. This host allowed more circumscribed conditions of the prevalence of *T. granarium* larvae at high temperatures and storage periods indicating particularly high future adult populations of this species, and the concomitant progeny production (**Figure 10**). Overall, based on the results of the present study, the numbers of larvae of *T. granarium* at 30 and 35°C were extremely high, and, at longer experimental intervals, *R. dominica* and *S. oryzae* populations rapidly collapsed.

## Discussion

Cereal host, temperature, and competing species were tested here regarding their potential impact on the establishment of *T. granarium*. As this invasive species is well-adapted to cereal grains and cereal products at warmer temperatures, its invasive potential should be higher at temperatures within the range of 30–35°C in both paddy rice and wheat (Banks, 1977; Finkelman et al., 2006; Hosseininaveh et al., 2007; Burges, 2008). In contrast, *R. dominica* is a species of temperate origin that adapted well to warmer climates in its world spread (Potter, 1935; Fields et al., 1993; Guedes et al., 1997), while *S. oryzae* is better adapted to small cereals and subtropical (mild) conditions (Corrêa et al., 2013, 2017). The expectations regarding *T. granarium* were proven misguided for the adult population of this species, but not so for its larvae confirming the significance of high temperatures for this species establishment. Hence, considering the overall numbers of *T. granarium* (i.e., adults and larvae), it becomes evident that this species can prevail over major grain beetles at elevated temperatures. This is particularly important, as larvae of this species are long-lived and contibute greatly to its spread; on the other hand, adults do not fly, and their contribution in “long-jump” invasions is limited. The large larval population recorded are the accumulated results of sucessive generations of the initial adults used in establishing the experimental units, as these adults do not leave long (usually, less than 2 weeks) exhibiting a peak in egg-laying within the 1st week after emergence laying over 50 eggs/female at the range of temperatures tested (European and Mediterranean Plant Protection Organization [EPPO], 1981; Burges, 2008; Stibick, 2009), and whose steady adult numbers are the result of replacement of the original insects by part of its (non-diapausing) progeny. The collapse of the adult population of *R. dominica* and *S. oryzae* at longer periods and high temperatures indicates that for these two species, larval numbers collapsed, although this was not measured as immature development occurs within the kernel. Hence, *T. granarium* can displace the other two species.

The overall response of *R. dominica* and *S. oryzae* to temperature was within the expectation with these species doing better at milder temperatures (Hill, 2003; Robinson, 2005; Mason and McDonough, 2012). The alleged good adaptation and performance expected for *T. granarium* at warmer temperatures were not apparent for the adults in our experiments in which the invasive *T. granarium* was outcompeted by both *R. dominica* and *S. oryzae*, particularly at the lower temperature range tested. Furthermore, both local competing species did better in wheat rather than paddy rice, including *S. oryzae*, although the competitive performance of *R. dominica* was higher in paddy rice than wheat, in stark opposition to *S. oryzae*. It has been recently established that *T. granarium* can develop much better in wheat than in rice (Athanassiou et al., 2016), as also observed in our study. However, most of the interceptions in United States are in rice (Myers and Hagstrum, 2012). This should be seriously taken into account, given that *T. granarium* remains in rice at low numbers and often undetected, and may easily build up high populations in wheat when the prevailing conditions are suitable, outperforming with great ease other major stored-product insect species.

The low prevalence of *T. granarium* at 25°C may not be due to its inferior competitiveness at these conditions, but due to its ability to induce (facultative) larva diapause when temperatures are below 29°C (Wilches et al., 2016). The low, but stable number of *T. granarium* individuals at 25°C, a number which was apparently unaffected by the other two species, provide support for this notion. In contrast, based on our data for *T. granarium* larvae, this species can easily build up very high populations in 2–3 successive generations outperforming the other two species tested without directly interfering with them. Thus, eventual reduction in *R. dominica* and *S. oryzae* numbers seems basically due to the temperature conditions above their suitable range of performance rather than to the direct interaction with *T. granarium*.

Even low populations of *T. granarium* can lead to outbreaks of this species. Evidence for this come from a study by Karnavar (1972) reporting that *T. granarium* females originated from diapausing larvae exhibit higher fecundity than those that originated from non-diapausing larvae, which will result in higher population growth (Banks, 1977). Although biased, we considered essential to use the larval numbers as the key indicator of the competition, since the proportion of larvae to the overall population of *T. granarium* was extremely high and probably higher than that for the other species, although quantification of larval infestation for them was not possible due to their development hidden within the grain kernel. In other words, *T. granarium* is short lived at the adult stage (ca. 2 weeks), and most of the lifespan is spent at the larval stage (30–100 days) (Burges, 2008; Stibick, 2009), while the reverse is true for the other two species (Aitken, 1975). In this regard, when the conditions are suitable (i.e., temperatures are 30°C or higher) the larvae of *T. granarium* are more numerous and have faster development than those of *R. dominica* and the *S. oryzae*. At these conditions, our study shows that differences in population growth of the species tested are due to the different thermal requirements, rather than direct competition patterns for the same food source.

Facultative larval diapause is an important trait of *T. granarium* as an adaptive strategy contributing to its quarantine status and perceived importance. Although an important adaptation to adverse conditions, diapause in *T. granarium* larva compromises its population growth under direct competition, which is a condition that elicits physiological arrest. As a consequence, the species population strives, but do not grow as its competitors that do not exhibit the same physiological arrest. Surprisingly, our results show that, in some of the combinations tested, the simultaneous presence of other species in the same vial was beneficial for the population growth of *T. granarium*. Previous studies show that the young larvae of *T. granarium* feed on the embryo and only large larvae are able to feed easily on the grain endosperm (Hadaway, 1955; Burges, 1960; Banks, 1977). Therefore, newly hatched larvae of *T. granarium* may benefit from the frass and/or cracked kernels produced by other co-occurring species. Recent findings by Athanassiou et al. (2016) provide evidence for the beneficial role of cracked kernels in the rapid population growth of *T. granarium*. In that study, the authors found that population growth of this species was more than three times higher on cracked kernels than in whole wheat kernels (Athanassiou et al., 2016).

The reported outcome of our study raises questions regarding the invasiveness potential of *T. granarium* and the invasibility of the cereal-producing countries where this species has not been introduced. *Trogoderma granarium* invasiveness potential may not be as serious as alleged based on its outperformance by *R. dominica* and *S. oryzae* suggesting difficulties in its establishment and spread, particularly under mild temperatures (i.e., ≤20°C). Furthermore, its economic impact may also be smaller than anticipated, a perception that is apparently reinforced by its relatively small economic impact in Europe (European and Mediterranean Plant Protection Organization [EPPO], 1981, 2013). Under this prism, the invasiveness of *T. granarium* and the invasibility of the southwestern United States, Neotropical America, and Australia should be quantitatively considered in current pest-risk analyses to reliably assess the importance of *T. granarium* as an invasive species and quarantine concern in these regions. In this effort, two further aspects need to be considered – the damage losses caused by *T. granarium* and difficulties for controlling this species (Hill, 2003; Paini and Yemshanov, 2012; Kavallieratos et al., 2016, 2017).

*Trogoderma granarium* was unable to displace its competing species at low temperatures, but it was not displaced by them either, although retaining minimal population growth and low population numbers of individuals. Thus, the co-existence among these species at these conditions seems likely and their damage to the grain will escalate, particularly considering that there is no apparent negative interaction among them. Our path analyses indicated positive association among these species (i.e., increase in numbers from each lead to increase in the others). The competing species do not seem to be directly interfering with one another, what is reinforced by their partitioning of the common resource explored (i.e., the grain kernels), but only reproductively outperforming each other, which may be more strongly expressed under conditions of reduced food availability. As far as we are aware, there is no evidence of direct larval interference and cannibalism in *T. granarium*, *R. dominica*, and *S. oryzae*, although this phenomenon has been reported in the related *Sitophilus zeamais* with implications for its fitness and competitive performance (Guedes et al., 2010).

As a “dirty feeder,” *T. granarium* enhances rapid grain contamination, compromising product palatability (Stibick, 2009). Again our path analyses reinforce this notion as the presence of *T. granarium* significantly increased frass production and reduced final grain weight (i.e., increased grain loss) much more than *R. dominica*. In fact, even in cases where conditions were not favorable for *T. granarium* population growth, this species was able to substantially compromise grain quality and enhance grain loss regardless of the competing species. This observation illustrates the potential of the larvae of *T. granarium* to cause serious infestations in a short period of time, even when larval numbers are not high. Also, this observation suggests that, despite its potentially reduced competitiveness and even invasiveness when faced with the *R. dominica* and *S. oryzae* at low temperatures, *T. granarium* sustains its grain loss capacity and likely its economic relevance.

The rapid destruction of the grain kernels by *T. granarium* larvae is expected to be highly detrimental for the population of *R. dominica* and *S. oryzae*, as these species, as internal feeders, require sound kernels for their development and progeny production (Aitken, 1975). This may be the key for the rapid decline of the populations of these two internal feeders when larval numbers of *T. granarium* are increasing. Hence, while *T. granarium* is not affected by the presence of the other two species, the other two species, under certain conditions, may be negatively affected by the simultaneous presence of *T. granarium* in the same habitat. Conversely, the previous infestation by *R. dominica* and *S. oryzae* may be beneficial for *T. granarium* development. Additional experimentation is required to examine further the basis of these hypotheses.

Eventual temperature increase will likely favor *T. granarium* through high progeny production and extremely rapid population growth. At these conditions, *T. granarium* prevails even in already heavily infested commodities by the other two species. In general, temperatures around 30°C, are considered normal for several months after harvest in bulked grains in the temperate zones throughout the globe (Athanassiou and Buchelos, 2001; Hagstrum and Subramanyam, 2009). Still, in rice, the outcome of the competition is more uncertain than in wheat. In this context, rice may serve as a non-preferred “vehicle” host for *T. granarium*, on which larvae remain undetected for long periods, due to their low numbers. The reduced chances of detection in rice may be also linked with diapause induction, which may explain the prevalence of interceptions in rice reported in the United States, and also in other countries (European and Mediterranean Plant Protection Organization [EPPO], 1981; Myers and Hagstrum, 2012).

One last aspect to consider regarding the invasive and quarantine importance of *T. granarium* is the management difficulties imposed by this species making its control rather difficult. The refuge seeking behavior of the species, together with its facultative diapause, are two noteworthy management aspects to consider. The near mature larvae of *T. granarium* usually leave their food and seek for refuge in crevices, making their control extremely challenging, especially in transport facilities (Bell and Wilson, 1995; Stibick, 2009). The own thigmotactic stimulation of a crevice (i.e., movement toward the crevice mechanical stimuli), in addition to adverse conditions will spark the developmental arrest of the larvae through facultative diapause increasing their chances of survival (Stibick, 2009; Wilches et al., 2016). Increased survival of this species to standard management tactics that are usually effective against other major stored-product pests (Bell and Wilson, 1995; Wilches et al., 2016), will also further compromise management, which may result in further spread of *T. granarium*. This is particularly important at longer storage periods, since our results show that, if there is no control of *T. granarium* in its initial population levels, after 2 months or more this species can become a problem for grains, much more than the other two species tested here.

## Conclusion

In summary, although outperformed under direct competition with local beetle species at moderate temperatures, *T. granarium* is not displaced and is able to sustain low adult populations in both wheat and paddy rice. Moreover, the large larval populations recorded at high temperatures later in the storage period clearly suggest potential future outbreaks. The population growth of this invasive species is temperature-dependent and does not seem directly impaired by its competitors. The results with adult population of *T. granarium* by themselves question the notion of the currently perceived importance of this species as a quarantine priority in the United States, Neotropical America, and Australia. However, the drastic increase in larval population at high temperatures, the grain loss caused by this species that seem to surpass that of some of its potential competitors, and the difficulties in controlling the species lay credence to the cautionary approach in recognizing its importance as invasive species. Overall, larvae of this species can easily dominate when conditions are suitable, utilizing as food sources already infested grains by its competitors, causing serious infestations. Fine-tuning of the opposite contribution of these traits will allow the recognition of the relevance and quarantine status of this pest, allowing suitable decision-making regarding the eventual mitigating measures necessary to deal with it.

## Author Contributions

NK and CA conceived and designed the study. NK, JD, and MB performed the research. NK, CA, and RG analyzed the data. NK, CA, and RG wrote the paper.

## Conflict of Interest Statement

The authors declare that the research was conducted in the absence of any commercial or financial relationships that could be construed as a potential conflict of interest.
