# Supplementary material for: Invader Competition with Local Competitors: Displacement or Coexistence among the Invasive Khapra Beetle, Trogoderma granarium Everts (Coleoptera: Dermestidae), and Two Other Major Stored-Grain Beetles?
Source: Front Plant Sci. 2017 Nov 7;8:1837. doi: 10.3389/fpls.2017.01837 (PMC5681968; doi:10.3389/fpls.2017.01837)
Supplement: Supplementary file 1 [file Table_1.DOC]

SUPPLEMENTARY TABLE 1. Four-way ANOVA parameters of the single-species experiment with number of adults as the response variable and commodity, insect species, temperature and time as the independent variables.

| **Source of variation** | **DF** | *F* | ***P*** |
| --- | --- | --- | --- |
| Commodity | 1 | 397.0 | < 0.01 |
| Insect species | 2 | 152.2 | < 0.01 |
| Temperature | 2 | 79.8 | < 0.01 |
| Time | 2 | 95.6 | < 0.01 |
| Commodity x insect species | 2 | 45.8 | < 0.01 |
| Commodity x temperature | 2 | 66.1 | < 0.01 |
| Commodity x time | 2 | 43.1 | < 0.01 |
| Insect species x temperature | 4 | 108.8 | < 0.01 |
| Insect species x time | 4 | 46.7 | < 0.01 |
| Temperature x time | 4 | 40.9 | < 0.01 |
| Commodity x insect species x temperature | 4 | 85.7 | < 0.01 |
| Commodity x insect species x time | 4 | 24.9 | < 0.001 |
| Insect species x temperature x time | 8 | 18.7 | < 0.001 |
| Commodity x insect species x temperature x time | 12 | 21.7 | < 0.001 |
| Error | 432 | - | - |

Supplementary Table 2. Two-way ANOVA parameters of the single-species experiment with frass produced and grain loss as the response variables and commodity and insect species as the independent variables (temperature and time were covariates).

| **Source of variation** | **DF** | Frass produced | | **Grain loss** | |
| --- | --- | --- | --- | --- | --- |
|  |  | **F** | ***P*** | **F** | ***P*** |
| Commodity | 1 | 218.4 | < 0.001 | 146.86 | < 0.01 |
| Insect species | 2 | 26.6 | < 0.001 | 20.56 | < 0.01 |
| Commodity x insect species | 2 | 15.9 | < 0.001 | 12.84 | < 0.01 |
| Temperature | 2 | 1.3 | 0.29 | 1.37 | 0.25 |
| Time | 2 | 42.2 | < 0.001 | 60.04 | < 0.01 |
| Error | 476 | - | - | - | - |

Supplementary Table 3. Three-way ANOVA parameters of two-species competition with number of adults as the response variable and commodity, temperature and time as the independent variables.

| **Source of variation** | **DF** | ***R. dominica - S. oryzae*** | | ***R. dominica - T. granarium*** | | ***S. oryzae - T. granarium*** | |
| --- | --- | --- | --- | --- | --- | --- | --- |
|  |  | **F** | ***P*** | **F** | ***P*** | **F** | ***P*** |
| Commodity | 1 | 14.9 | < 0.01 | 4.5 | 0.03 | 482.9 | < 0.01 |
| Temperature | 2 | 42.5 | < 0.01 | 82.9 | < 0.01 | 489.5 | < 0.01 |
| Time | 2 | 12.9 | < 0.01 | 16.4 | < 0.01 | 7.9 | < 0.01 |
| Commodity x temperature | 2 | 11.9 | < 0.01 | 17.0 | < 0.01 | 411.7 | < 0.01 |
| Commodity x time | 2 | 4.1 | 0.02 | 8.3 | < 0.01 | 7.2 | 0.001 |
| Temperature x time | 4 | 4.5 | < 0.01 | 26.5 | < 0.01 | 11.8 | < 0.01 |
| Commodity x temperature x time | 4 | 0.8 | 0.50 | 7.7 | < 0.01 | 8.3 | < 0.01 |
| Error | 161 | - | - | - | - | - | - |

Supplementary Table 4. Two-way ANOVA parameters of two-species competition with frass produced and grain loss as the response variables and commodity and insect species as the independent variables (temperature and time were covariates).

| **Source of variation** | **DF** | **Frass produced** | | **Grain loss** | |
| --- | --- | --- | --- | --- | --- |
|  |  | **F** | ***P*** | **F** | ***P*** |
| Commodity | 1 | 385.8 | < 0.01 | 294.9 | < 0.01 |
| Insect species | 2 | 21.7 | < 0.01 | 11.7 | < 0.01 |
| Commodity x insect species | 2 | 20.9 | < 0.01 | 20.4 | < 0.01 |
| Temperature | 2 | 2.0 | 0.14 | 8.9 | < 0.01 |
| Time | 2 | 66.1 | < 0.01 | 73.7 | < 0.01 |
| Error | 476 | - | - | - | - |

Supplementary Table 5. Three-way ANOVA parameters of three-species competition with number of adults as the response variable and commodity, temperature and time as the independent variables.

| **Source of variation** | **DF** | ***R. dominica*** | | ***S. oryzae*** | | ***S. oryzae*** | | **Frass produced** | | **Grain loss** | |
| --- | --- | --- | --- | --- | --- | --- | --- | --- | --- | --- | --- |
|  |  | **F** | ***P*** | **F** | ***P*** | **F** | ***P*** | **F** | ***P*** | **F** | ***P*** |
| Commodity | 1 | 32.0 | < 0.01 | 1327.9 | < 0.01 | 147.9 | < 0.01 | 2647.4 | < 0.01 | 3844.9 | < 0.01 |
| Temperature | 2 | 106.4 | < 0.01 | 1528.9 | < 0.01 | 14.8 | < 0.01 | 4.9 | < 0.01 | 68.9 | < 0.01 |
| Time | 2 | 5.5 | < 0.01 | 97.8 | < 0.01 | 101.7 | < 0.01 | 555.8 | < 0.01 | 1073.2 | < 0.01 |
| Commodity x temperature | 2 | 6.1 | < 0.01 | 1353.5 | < 0.01 | 26.8 | < 0.01 | 10.3 | < 0.01 | 94.5 | < 0.01 |
| Temperature x time | 2 | 2.1 | 0.13 | 91.2 | < 0.01 | 95.1 | < 0.01 | 556.0 | < 0.01 | 872.9 | < 0.01 |
| Temperature x time | 4 | 20.4 | < 0.001 | 115.8 | < 0.01 | 39.6 | < 0.01 | 16.5 | < 0.01 | 48.6 | < 0.01 |
| Commodity x temperature x time | 4 | 8.6 | < 0.001 | 105.1 | < 0.01 | 35.1 | < 0.01 | 17.3 | < 0.01 | 49.9 | < 0.01 |
| Error | 161 | - | - | - | - | - | - | - | - | - | - |

**SUPPLEMENTARY TABLE 6.** **Effect of storage time and temperature on the adult population of three competing species in two commodities.**

| **Period** |  | **65 days** |  |  | **130 days** |  |  | **200 days** |  | ***F*** | ***P*** |
| --- | --- | --- | --- | --- | --- | --- | --- | --- | --- | --- | --- |
|  | **25^o^C** | **30^o^C** | **35^o^C** | **25^o^C** | **30^o^C** | **35^o^C** | **25^o^C** | **30^o^C** | **35^o^C** |  |  |
| Initial species  in the vial |  |  |  |  |  |  |  |  |  |  |  |
| *R. dominica*  (paddy rice) | 15.0 ± 0.0 Fb | 22.2 ± 2.0 EFc | 26.0 ± 2.3 EFb | 37.1 ± 5.2 DEFc | 43.6 ± 4.2 CDEb | 50.6 ± 5.6 BCDb | 63.9 ± 9.6 BCc | 109.8 ± 4.7 Ab | 69.9 ± 7.8 Bb | 30.2 | < 0.01 |
| *R. dominica*  (wheat) | 17.2 ± 0.9 Cb | 56.3 ± 8.5 BCb | 69.9 ± 14.9 BCa | 106.9 ± 27.5 BCb | 172.4 ± 44.6 ABa | 159.0 ± 40.6 ABCa | 272.4 ± 16.9 Ab | 252.3 ± 14.1 Aa | 190.2 ± 61.0 ABa | 7.8 | < 0.01 |
| *S. oryzae*  (paddy rice) | 25.7 ± 1.3B Cb | 18.8 ± 1.0 CDc | 15.2 ± 0.2 Db | 32.3 ± 2.2 Bc | 31.4 ± 1.8 Bb | 14.7 ± 0.5 Db | 48.1 ± 4.3 Acd | 15.0 ± 0.0 Dc | 15.0 ± 0.0 Db | 40.0 | < 0.01 |
| *S. oryzae*  (wheat) | 86.6 ± 21.4 Ca | 21.7 ± 2.4 DEc | 15.0 ± 0.0 Eb | 323.6 ± 19.0 Ba | 70.6 ± 9.4 CDb | 15.1 ± 0.3 Eb | 632.3 ± 16.3 Aa | 14.9 ± 0.5 Ec | 14.6 ± 0.3 Eb | 343.9 | < 0.01 |
| *T. granarium*  (paddy rice) | 13.0 ± 1.0 Ab | 7.3 ± 2.4 BCc | 2.9 ± 1.0 CDb | 9.6 ± 1.0 ABc | 1.7 ± 0.6 Db | 0.3 ± 0.3 Db | 11.8 ± 0.9 ABd | 4.0 ± 1.2 CDc | 0.0 ± 0.0 Db | 19.5 | < 0.01 |
| *T. granarium*  (wheat) | 16.1 ± 1.1 Deb | 86.8 ± 9.7 Aa | 70.3 ± 13.4 ABa | 14.2 ± 2.5 Dec | 49.1 ± 6.7 BCb | 0.3 ± 0.2 Eb | 30.2 ± 1.9 CDcd | 7.2 ± 2.8 Dec | 2.0 ± 1.2 Eb | 26.0 | < 0.01 |
| *F* | 10.6 | 29.7 | 12.8 | 76.0 | 9.7 | 13.3 | 532.4 | 255.6 | 8.6 |  |  |
| *P* | < 0.01 | < 0.01 | < 0.01 | < 0.01 | < 0.01 | < 0.01 | < 0.01 | < 0.01 | < 0.01 |  |  |

*Within each row, means followed by the same uppercase latter are not significantly different (P < 0.05, DF* *= 8, 80; with Tukey Kramer honestly significant difference test; SAS Institute, Cary, NC, USA). Within each column, means followed by the same lowercase letter, are not significantly different (P < 0.05, DF = 5, 53; with Tukey Kramer honestly significant difference test; SAS Institute, Cary, NC, USA)*.

**SUPPLEMENTARY TABLE 7.** **Effect of storage time and temperature on the larva population of *T. granarium* in two commodities under different competition scenarios.**

| **Period** |  | **65 days** |  |  | **130 days** |  |  | **200 days** |  | ***F*** | ***P*** |
| --- | --- | --- | --- | --- | --- | --- | --- | --- | --- | --- | --- |
|  | ***R. dominica*** | ***S. oryzae*** | ***T. granarium*** | ***R. dominica*** | ***S. oryzae*** | ***T. granarium*** | ***R. dominica*** | ***S. oryzae*** | ***T. granarium*** |  |  |
| Initial species  in the vial |  |  |  |  |  |  |  |  |  |  |  |
| *T. granarium*  (25^o^C - paddy rice) |  |  | 37.6 ± 6.4 d |  |  | 29.1 ± 2.8 f |  |  | 21.2 ± 5.3 e | 2.6 | 0.09 |
| *T. granarium*  (25^o^C - wheat) |  |  | 101.9 20.3 Ad |  |  | 43.0 ± 7.4 Bf |  |  | 53.1 ± 4.3 Be | 6.2 | 0.01 |
| *T. granarium*  (30^o^C - paddy rice) |  |  | 67.5 ± 8.7 Ad |  |  | 37.8 ± 4.3 Bf |  |  | 62.8 ± 7.7 Abe | 5.1 | 0.02 |
| *T. granarium*  (30^o^C - wheat) |  |  | 687.3 ± 58.4 Cb |  |  | 1195.1 ± 50.9 Bd |  |  | 2283.0 ± 66.7 Ac | 190.8 | < 0.01 |
| *T. granarium* (35^o^C - paddy rice) |  |  | 52.2 ± 6.7 d |  |  | 58.2 ± 4.7 f |  |  | 57.4 ± 5.9 e | 0.3 | 0.74 |
| *T. granarium*  (35^o^C - wheat) |  |  | 1124.2 ± 97.4 Ca |  |  | 2278.0 ± 85.5 Bb |  |  | 3659.1 ± 30.9 Ab | 272.3 | < 0.01 |
| *T. granarium* x *R. dominica* (25^o^C - paddy rice) | 21.0 ± 1.3 BCcd |  | 25.8 ± 3.3 Bd | 51.0 ± 1.9 Abc |  | 5.6 ± 1.3 Cf | 64.8 ± 7.7 Ab |  | 23.6 ± 3.4 Be | 32.3 | < 0.01 |
| *T. granarium* x *R. dominica* (25^o^C - wheat) | 14.4 ± 1.8 Ccd |  | 81.0 ± 14.6 BCd | 93.6 ± 12.5 Ba |  | 181.9 ± 11.9 A | 140.1 ± 23.1 ABa |  | 193.0 ± 27.6 Ae | 15.2 | < 0.01 |
| *T. granarium* x *R. dominica* (30^o^C - paddy rice) | 28.4 ± 7.1 Babc |  | 17.1 ± 3.2 Bd | 69.3 ± 2.9 Aab |  | 31.2 ± 5.4 Bf | 21.8 ± 8.2 Bcd |  | 65.0 ± 6.3 Ae | 15.0 | < 0.01 |
| *T. granarium* x *R. dominica* (30^o^C - wheat) | 39.9 ± 7.5 Dab |  | 407.9 ± 25.9 Cc | 26.4 ± 7.3 Dcde |  | 1859.6 ± 134.0 Bc | 29.8 ± 3.4 Dbcd |  | 2513.3 ± 165.0 Ac | 156.2 | < 0.01 |
| *T. granarium* x *R. dominica* (35^o^C - paddy rice) | 12.1 ± 3.6 Ccd |  | 47.0 ± 3.4 Bd | 19.7 ± 4.8 Cdef |  | 43.6 ± 5.7 Bf | 0.8 ± 0.4 Cd |  | 71.3 ± 9.5 Ae | 24.3 | < 0.01 |
| *T. granarium* x *R. dominica* (35^o^C - wheat) | 12.4 ± 2.0 Dcd |  | 546.4 ± 29.4 Cbc | 0.1 ± 0.1 Df |  | 3111.2 ± 28.2 Ba | 0.6 ± 0.4 Dd |  | 3962.1 ± 72.4 Aab | 2778.0 | < 0.01 |
| *T. granarium* x *S. oryzae* (25^o^C - paddy rice) |  | 19.0 ± 1.4 Bcd | 19.2 ± 3.3Bd |  | 23.9 ± 2.0 Bcd | 18.7 ± 3.3 Bf |  | 35.6 ± 3.1 Ac | 17.1 ± 1.9 Be | 7.0 | < 0.01 |
| *T. granarium* x *S. oryzae* (25 ^o^C - wheat) |  | 338.6 ± 15.4 Ba | 14.1 ± 2.1 Cd |  | 512.3 ± 38.6 Aa | 49.1 ± 11.6 Cf |  | 520.7 ± 36.4 Aa | 25.6 ± 5.7 Ce | 110.4 | < 0.01 |
| *T. granarium* x *S. oryzae* (30^o^C - paddy rice) |  | 11.4 ± 1.1 Ccd | 26.6 ± 4.3 BCd |  | 12.6 ± 1.4 BCcd | 35.7 ± 6.0 Bf |  | 4.2 ± 0.8 Cc | 113.1 ± 11.6 Ae | 51.0 | < 0.01 |
| *T. granarium* x *S. oryzae* (30^o^C - wheat) |  | 35.2 ± 4.7 Dc | 438.1 ± 24.5 BCc |  | 66.8 ± 27.6 CDc | 561.7 ± 185.5 Be |  | 0.0 ± 0.0 Dc | 2225.7 ± 107.1 Ac | 91.9 | < 0.01 |
| *T. granarium* x *S. oryzae* (35^o^C - paddy rice) |  | 7.8 ± 0.9 Cd | 37.4 ± 3.9 Bd |  | 6.4 ± 0.7 Ccd | 61.0 ± 5.0 Af |  | 1.7 ± 0.5 Cc | 68.9 ± 2.8 Ae | 107.2 | < 0.01 |
| *T. granarium* x *S. oryzae* (35^o^C - wheat) |  | 13.0 ± 0.8 Dcd | 1064.4 ± 58.8 Ca |  | 0.0 ± 0.0 Dd | 3102.4 ± 31.7 Ba |  | 0.0 ± 0.0 Dc | 4083.0 ± 129.0 Aa | 913.2 | < 0.01 |
| *T. granarium* x *R. dominica* x *S. oryzae* (25^o^C - paddy rice) | 22.8 ± 2.1 Bbcd | 15.1 ± 0.5 Bcd | 26.2 ± 7.3 Bd | 17.3 ± 1.5 Bdef | 21.7 ± 1.7 Bcd | 19.2 ± 4.2 Bf | 47.3 ± 5.3 Abc | 18.6 ± 0.7 Bc | 21.4 ± 3.1 Be | 7.0 | < 0.01 |
| *T. granarium* x *R. dominica* x *S. oryzae* (25^o^C - wheat) | 13.6 ± 1.6 Fcd | 128.9 ± 7.1 Cb | 82.2 ± 5.8 Dd | 49.2 ± 5.8 Ebc | 289.9 ± 9.3 Bb | 19.9 ± 3.5 EFf | 51.2 ± 5.5 DEbc | 338.9 ± 13.2 Ab | 21.1 ± 4.3 EFe | 300.8 | < 0.01 |
| *T. granarium* x *R. dominica* x *S. oryzae* (30^o^C - paddy rice) | 20.3 ± 2.6 Bcd | 12.9 ± 0.5 Bcd | 20.1 ± 1.8 Bd | 18.4 ± 3.2 Bdef | 14.8 ± 0.3 Bcd | 47.7 ± 5.9 Af | 18.3 ± 3.9 Bcd | 11.6 ± 0.9 Bc | 57.7 ± 4.9 Ae | 25.2 | < 0.01 |
| *T. granarium* x *R. dominica* x *S. oryzae* (30^o^C - wheat) | 41.4 ± 5.3 Ca | 18.7 ± 1.5 Ccd | 568.6 ± 18.8Bbc | 30.8 ± 6.0 Ccd | 16.2 ± 3.5 Ccd | 551.9 ± 13.4 Be | 35.7 ± 4.7 Cbcd | 14.3 ± 3.1 Cc | 661.4 ± 25.2 Ad | 575.6 | < 0.01 |
| *T. granarium* x *R. dominica* x *S. oryzae* (35^o^C - paddy rice) | 8.3 ± 2.3 Cd | 9.8 ± 1.1 Cd | 47.9 ± 7.4 Bd | 2.1 ± 0.7 Cef | 9.7 ± 1.1 Ccd | 62.8 ± 6.0 ABf | 1.0 ± 0.4 Cd | 8.8 ± 0.7 Cc | 66.3 ± 2.8 Ae | 60.7 | < 0.01 |
| *T. granarium* x *R. dominica* x *S. oryzae* (35^o^C - wheat) | 16.6 ± 1.2 Dcd | 9.1 ± 1.1 Dd | 1107.1 ± 56.3 Ca | 0.7 ± 0.5 Df | 2.1 ± 0.8 Dcd | 3250.0 ± 82.5 Ba | 2.2 ± 1.0 Dd | 2.7 ± 1.2 Dc | 3940.4 ± 162.4 Aab | 606.8 | < 0.01 |
| *F* | 7.9 | 344.1 | 148.6 | 31.1 | 126.5 | 423.3 | 24.8 | 223.7 | 595.0 |  |  |
| *P* | < 0.01 | < 0.01 | < 0.01 | < 0.01 | < 0.01 | < 0.01 | < 0.01 | < 0.01 | < 0.01 |  |  |

*Within each row, means followed by the same uppercase letter are not significantly different (T. granarium: P < 0.05, DF = 2, 26; T. granarium x R. dominica, T. granarium x S. oryzae: P < 0.05, DF = 5, 53; T. granarium x R. dominica x S. oryzae: P < 0.05, DF = 8, 80; Tukey Kramer honestly significant difference test; SAS Institute, Cary, NC, USA). Within each column, means followed by the same lowercase letter, are not significantly different (R. dominica, S. oryzae: P < 0.05, DF = 11, 107; T. granarium: P = 0.05, DF = 23, 215; with Tukey Kramer honestly significant difference test; SAS Institute, Cary, NC, USA).*
